# Facilitating transition between adolescent and adult psychiatry: patient and parent experiences from a jointly run ward for 16-25-year-olds in Germany

**DOI:** 10.1186/s13034-026-01059-8

**Published:** 2026-03-14

**Authors:** Lena-Sophie Over-Pluschke, Annette Duve, Sevinc Jürgens, Sebastian von Peter, Julian Schwarz

**Affiliations:** 1 Department of Psychiatry and Psychotherapy, Center for Mental Health, Immanuel Hospital Rüdersdorf, Brandenburg Medical School Theodor Fontane, Seebad 82/83, 15562 Rüdersdorf, Germany; 2 Faculty of Health Sciences Brandenburg, Brandenburg Medical School Theodor Fontane, Neuruppin, Germany; 3 Vitos Children’s and Adolescent Clinic for Mental Health, Riedstadt, Germany

**Keywords:** Transition, Adolescent psychiatry, Needs assessment, Flexible and integrative care, Acute outpatient treatment

## Abstract

**Background:**

Young people in the transition from adolescence to adulthood face particular challenges in psychiatric care. Continuity of treatment is often disrupted, as services are typically divided between child and adolescent psychiatry and adult psychiatry. Few frameworks provide age-appropriate, integrated care for individuals aged 16–25. In Germany, a model project was established to address this gap. It offers treatment tailored to this age group within a cross-sector, integrated care model that combines outpatient and inpatient services under a jointly run ward staffed by both child and adolescent psychiatrists and adult psychiatrists. This study explores the distinctive features of this integrated adolescent ward and examines the experiences of patients and their parents.

**Methods:**

Semi-structured interviews were conducted with 27 patients and 7 parents. Data were analyzed using qualitative content analysis, focusing on three domains: (1) framework conditions and access to care, (2) the concept and content of treatment, and (3) the environment of the integrated adolescent ward.

**Results:**

Participants particularly valued the individualized therapeutic approach, flexible treatment across different settings, and transparent, responsive communication by staff. Age homogeneity and a focus on adolescence facilitated mutual identification and supportive peer interactions. Opportunities for self-determination and participation were also emphasized as important.

**Conclusion:**

Findings suggest that specific structural elements contribute to the ward being perceived as an age-appropriate therapeutic environment. Further research should identify and define specific care components of integrated treatment for young people aged 16–25 to enable replication and evaluation of its implementation.

## Background

Growing international evidence shows that the demand for and utilization of psychiatric care among adolescents and young adults have increased substantially in the last decade [1, 2]. In the United States, for instance, the number of psychiatric emergency department visits among youth increased by 28% between 2011 and 2015 [3]. This upward trend was further exacerbated by the COVID-19 pandemic, which profoundly affected the mental health of young adults worldwide and underscored the need for age-specific services [4, 5]. The pandemic thus acted both as a magnifying lens for preexisting gaps in care and as an amplifier of these deficits, which remain evident internationally [6].

Adolescence is inconsistently defined in the literature; terms such as “young adults” or “youth” generally refer to individuals between 11 and 25 years of age [7]. This developmental period is considered a transitional phase in which identity, independence, and key life decisions are shaped, often referred to as “transition” [7]. It is well documented that the first mental disorder occurs before the age of 25 in 62.5% of affected individuals [8]. Early-onset disorders are associated with high morbidity and mortality; suicide is the third most common cause of death among 15- to 29-year-olds worldwide [9]. Young people with mental illness often exhibit deficits in social skills and coping strategies, making it more difficult to achieve key developmental milestones and increasing the risk of chronic conditions [10–12]. Mental disorders contribute substantially to the global burden of disease and have profound societal and economic implications [13]. In addition, there is an empirically proven correlation between mental illness and negative life events, such as unemployment [14]. The treatment of this age group is currently divided between child and adolescent psychiatry (CAP) and adult psychiatry (AP). Upon reaching the age of 18, patients transition from CAP, which is specialized in developmental tasks, to AP, where the episodic course of psychiatric disorders is the primary focus [15–17]. Neither of the two systems adequately addresses the specific needs of 16- to 25-year-olds. In CAP, the joint treatment of adolescents with much younger patients can foster regressive tendencies and stagnation, whereas in AP the lack of a pedagogical framework often increases the potential for conflicts and rule violations [18].

As a result, treatment interruptions of more than three months frequently occur during the transition phase, affecting about 75% of patients according to one study [19]. Such interruptions are considered a risk factor for further crises and hinder the provision of continuous, age-appropriate care [19–21]. International evidence shows that effective treatment during this critical life stage can significantly improve the long-term prognosis of mental illness and support the achievement of essential developmental milestones [11, 22, 23]. This underscores the urgency of early intervention [11, 12, 23]. At the same time, this age group makes less use of psychiatric services than others. The rising prevalence of mental disorders, coupled with low utilization of psychosocial services, highlights a substantial care gap for young adults [12, 24, 25]. Some countries have developed initial solutions to address these transitional challenges. In Australia, the “Headspace Centers” were established to reduce barriers to access and to provide low-threshold, holistic health care for 12- to 25-year-olds, aiming to intervene early in the development of mental illness [26]. Canada developed the “Foundry Centers,” which bring together central services for 12- to 24-year-olds and their families, including substance use care, physical and sexual health services, peer support, and social services [27]. Within the European Union, the results of the MILESTONE study are worth mentioning [28]. The study examines how a structured transition tool influences treatment success. Managed transition has a slightly positive effect on treatment success. All of these programs serve primarily as (digital) interfaces rather than as independent treatment options. Thus, what remains missing is a treatment model specifically tailored to this age group in the sense of adolescent psychiatry (ADO).

Despite the clear need for change, only a few specialized treatment programs for adolescents currently exist, and evaluation results regarding their effectiveness are rare [19–21, 29]. Only a small number of studies have systematically explored the experiences of patients and parents during the transition process [24, 30, 31]. An experience-based evaluation is therefore essential to inform the development of best-practice criteria for quality-oriented and standardized replication of existing care models.

The aim of this study is to evaluate an innovative, jointly operated ward for adolescents and adults that is co-managed by both CAP and AP to facilitate the transition between services. The perspectives of patients and their parents were included. Accordingly, this study addresses the following research question: How do patients and parents experience a flexible, integrative, cross-setting psychiatric care model designed for individuals aged 16 to 25 years?

## Methods

### Study design

The present paper reports the qualitative findings of a larger project evaluating a jointly run psychiatric ward for 16- to 25-year-olds within the framework of the EvAdoR study [32]. The experiences of patients and their parents are best captured through qualitative methods. To ensure quality, the COREQ checklist (“COnsolidated criteria for REporting Qualitative research”) was applied (see App. Table 1) [33]. Data was collected and analyzed by Brandenburg Medical School on behalf of the Vitos Klinikum Riedstadt. A contract for joint data use was established, and the responsible ethics committee of the state of Hesse approved the project on November 14, 2022.

### Setting

**Fig. 1:**
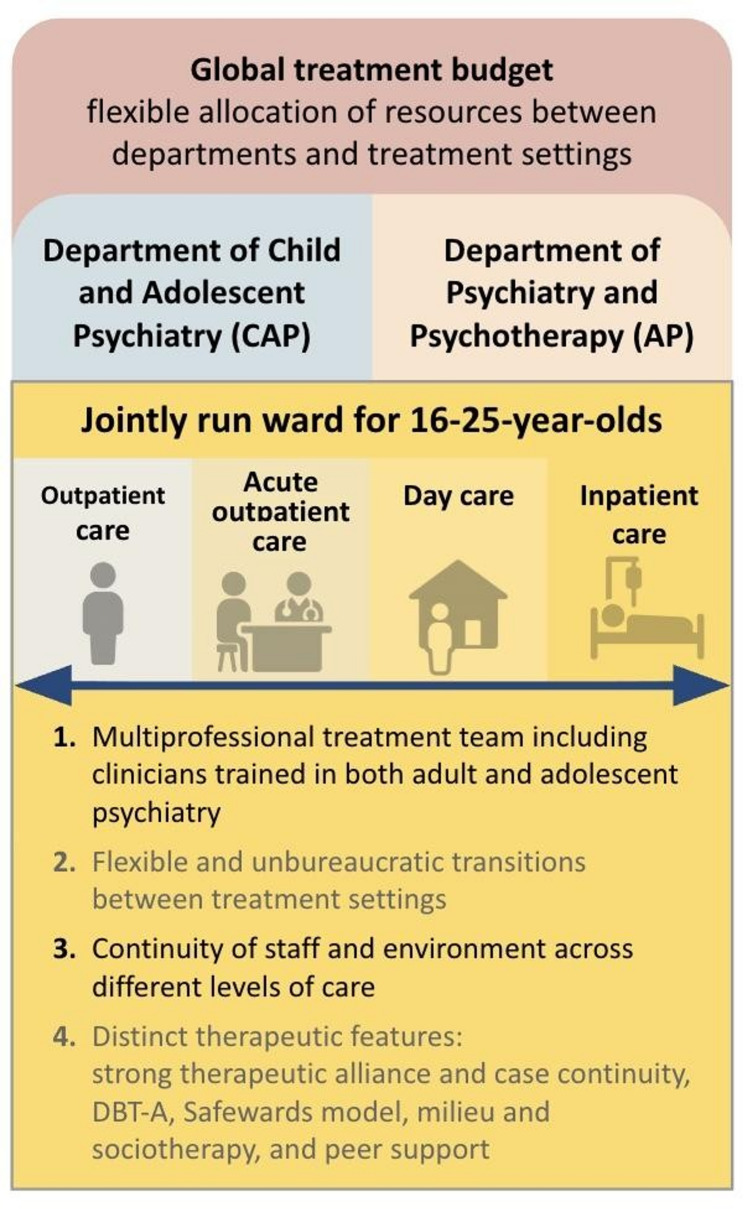
Visualisation of the jointly run psychiatric ward for 16–25-year-olds, including its key components. *n *Number of treatment places, *CAP *Child and Adolescent Psychiatry, *AP *Adult Psychiatry, *DBT-A *dialectic-behavioral-therapy for adolescents

The study was conducted in Germany, where CAP and AP usually exist separately in routine care. The investigated flagship project is among the first facilities in Germany to establish a jointly run ward for 16- to 25-year-olds and thus serves as a pioneering model see Fig. 1. The basis of this model was a global treatment budget negotiated with nearly all statutory health insurance providers. To date, there are 22 model projects for adult psychiatry and 5 in child and adolescent psychiatry based on § 64b SGB V (SGB = the central law governing statutory health insurance in Germany. However, none of the projects has implemented a cross-setting approach that is as advanced as the one in Riedstadt which enables flexible allocation of resources between departments and treatment settings and to implement community-based alternatives to inpatient care [34–38]. Cross-setting treatment refers to flexible transitions between inpatient, day hospital, and outpatient settings within one facility, adjusted to patient needs to avoid treatment discontinuities [39].

The ward was established under the joint leadership of CAP and AP and offers cross-sector treatment for young people aged 16 to 25. Professionals from both CAP and AP work in an integrated manner. The setting can be flexibly adjusted between 18 inpatient and day hospital places and 4 places in acute outpatient care (AOC).

- Inpatient: full stay including overnight stays.
- Day care: daytime participation in treatment offers without overnight stays.
- AOC: attendance only for scheduled treatment appointments, e.g., to initiate treatment, for stress testing, or for crisis intervention connected to the AP treatment episode.
- Outpatient care: attendance only for scheduled treatment appointments, e.g. for the initiation or follow-up of inpatient treatment, for crisis intervention or for indicated preventive support, including assessment of indications and advice on further assistance available within the framework of adolescent-specific developmental tasks detached from the AP treatment episode.

In total, there are 20 treatment places at ADO, which are adapted to the needs of the patients. As a rule, patients were treated in 2 AOC, 2 daycare, and 16 inpatient places. All settings are located within the same ward. Unlike in routine care, transitions between settings occur quickly and without bureaucracy, typically within a few days. This ensures stability of the framework, without the need for formal discharge letters or changes of treatment rooms or staff, while only the intensity of care is adjusted.

The treatment concept includes milieu and sociotherapy, primary nursing, de-escalation strategies, Dialectical behavioral therapy for adolescents (DBT-A), and Safewards [40–45]. As part of a sponsorship system based on Safewards, new patients are accompanied by more experienced peers during their first steps. In addition, a primary nursing system, consisting of one psychologist and two nurses, ensured continuity of care by providing fixed contact persons. Common treatment goals included support in the process of becoming independent, promotion of autonomy, coping with detachment problems, identity formation, socio-emotional maturation, and the development of practical everyday skills.

Patients under the age of 18 were admitted when chronic psychiatric disorders indicated a need for continued treatment into adulthood. For patients over 18, admission was based on socio-emotional development lagging behind biological age, thus requiring special support. All psychiatric disorders were treated, except for acute florid psychosis or acute risk to self or others.

### Sampling

A convenience sampling approach was used. All patients at the ADO were informed about the study and invited to participate. The sample specifically included those with experience both at the ADO in Riedstadt and in regular care.

Inclusion and exclusion criteria for patients were sufficient German language skills to participate in an interview, age between 16 and 25 years at the time of participation, presence of an ICD-10 F diagnosis, and no acute psychosis or acute risk of harm to self or others.

For parents, inclusion required living in the same household as the patient to ensure familiarity with daily challenges and to enable an assessment of the impact of treatment. Parents were excluded if they were experiencing acute strain or lacked sufficient German language skills.

### Data collection

Data was collected between April 2023 and April 2024. Based on a review of the relevant literature, semi-structured interview guides were developed (App. Table 2, App. Table 3) [54], drawing on existing research on psychiatric care for adolescents and identified knowledge gaps, and conceptually informed by Donabedian’s structure–process–outcome model to capture patients’ and parents’ experiences with structural conditions, care processes, and perceived outcomes of the newly implemented care model [55, 56]. Adolescents were recruited on site at the ADO by a trained researcher. Interviews were digitally recorded, transcribed, and anonymized. They were conducted either in person during the inpatient stay or by telephone about two weeks after discharge. To facilitate follow-up interviews, contact information was collected by a staff member on site. Written informed consent was obtained from patients, while parents gave consent digitally. In addition to interview data, sociodemographic information was collected. Field notes were taken during interviews and site visits to document contextual information and researcher impressions [57]. Field notes were taken to ensure the quality of the data, but were not analyzed as an independent data source. Participants received a compensation of €50 for their time and contributions. The interviews were conducted until data saturation was achieved.

### Data analysis

Data was analyzed using summarizing qualitative content analysis according to Mayring [58]. The aim was to systematically reduce the material while preserving essential content and to create a manageable, representative corpus through abstraction. Analysis was conducted using the software MAXQDA [59].

A coding system combining deductive and inductive elements was developed. The themes were generated deductively based on the themes in the interview guide (i.e., the implementation of critical components of the model or care, such as the atmosphere on the ward, the perception of the treatment team, the involvement of parents, and continuity of care), for which relevant literature was studied. The sub-themes emerged inductively from the data. Donabedian’s structure–process–outcome model was also used during data analysis as an analytic lens to organize and relate the inductively derived themes across structural conditions, care processes, and perceived outcomes [55, 56]. Data collection and analysis were conducted iteratively to fill knowledge gaps until thematic saturation was reached [60].

To ensure scientific rigor, interpretations were continuously scrutinized and reflected upon within the research perspective. A double-coding approach was applied: two researchers independently coded selected text passages, and discrepancies were discussed and resolved by consensus. In addition, regular team meetings, including senior staff of the ADO, were held to review and validate central themes, interpretations, and methodological decisions.

Declarations.

## Results

### Participants

During the survey period, 83 patients (April 2023 and April 2024) were treated at the ADO and invited to participate. A total of 26 patients treated at the ADO Riedstadt (32,5%) and 7 parents of these patients were interviewed. None of the participants included withdrew their participation after the fact. Fifteen interviews were conducted on site, while 19 were conducted by telephone. At the time of data collection, 76.9% of patients (*n* = 20) were in inpatient treatment, 19.2% (*n* = 5) in AOC, and 3.9% (*n* = 1) in day hospital treatment. The average length of stay was 9.5 weeks (SD = 3.37; range 3–15 weeks). In the six months prior to the current treatment, 26.9% (*n* = 7) had contact with outpatient CAP and 26.9% (*n* = 7) with outpatient AP. Prior inpatient experiences were reported by 3.9% (*n* = 1) in CAP and 7.7% (*n* = 2) in AP. Two patients (7.7%) had presented to a psychiatric emergency service, while 26.9% (*n* = 7) had received no psychological or psychiatric help. Psychopharmacological treatment was used by 92.3% (*n* = 24). Previous treatment experiences were reported with CAP (73.1%; *n* = 19), with AP (38.5%; *n* = 10), and with outpatient care (88.5%; *n* = 23). Earlier treatment discontinuations were reported by 19.2% (*n* = 5). Access to the current treatment was most often via referrals from office-based psychiatrists or psychotherapists (57.7%; *n* = 15). Additional pathways included transfers (11.5%), general practitioners (7.7%), emergency contacts (7.7%), self-referrals (7.7%), and advice from the social environment (3.9%).

The sociodemographic characteristics of the sample are presented in Table 1 (patients) and Table 2 (parents). In addition, sociodemographic descriptors were added for quotations in the appendix (App. Table 2).

**Table 1:** Patients’ sociodemographic data (*n* = 26)

Parameter	Value
Age	
M (SD)	19.35 (1,38)
min.-max.	17–23
Gender	n (%)
Female	15 (57,69)
Male	10 (38,46)
Non-binary	1 (3,85)
Housing situation	n (%)
With legal guardians / parents	22 (84,62)
Residential group	3 (11,54)
Supported individual living	1 (3,85)
Ethnicity	n (%)
White	26 (100,00)
Own children	n (%)
None	25 (96,15)
One child	1 (3,85)
Parents’ educational qualifications	n (%)
University degree	7 (26,92)
A-levels	15 (57,69)
Intermediate maturity	21 (80,77)
Secondary school	9 (34,62)
Own educational qualification	n (%)
University degree	0 (0,00)
A-levels	6 (23,08)
Intermediate maturity	15 (57,69)
Secondary school	5 (19,23)
Employment situation	n (%)
Not gainfully employed	4 (15,38)
School	16 (61,54)
Study	2 (7,69)
In training	2 (7,69)
Rehab measure	1 (3,85)
Voluntary year of social service	1 (3,85)

*M* Median, *SD* standard deviation, *n* sample size, *min.-max.* minimum to maximum

**Table 2:** Parents’ sociodemographic data

Parameter	Value
Age	
M (SD)	47,14 (4,41)
Gender	n (%)
Female	6 (85,71)
Male	1 (14,29)
Non-binary	0 (0,00)
Relationship to patient	n (%)
Father	1 (14,29)
Mother	6 (85,71)
Employment status	n (%)
Full-time	7 (100,00)
Number of own children	n (%)
M (SD)	1,57 (0,53)
min.-max.	1–2
Ethnicity	n (%)
White	7 (100,00)
Psychiatric diagnosis	n (%)
Yes	2 (28,57)
No	5 (71,43)

### Qualitative findings

**Fig. 2:**
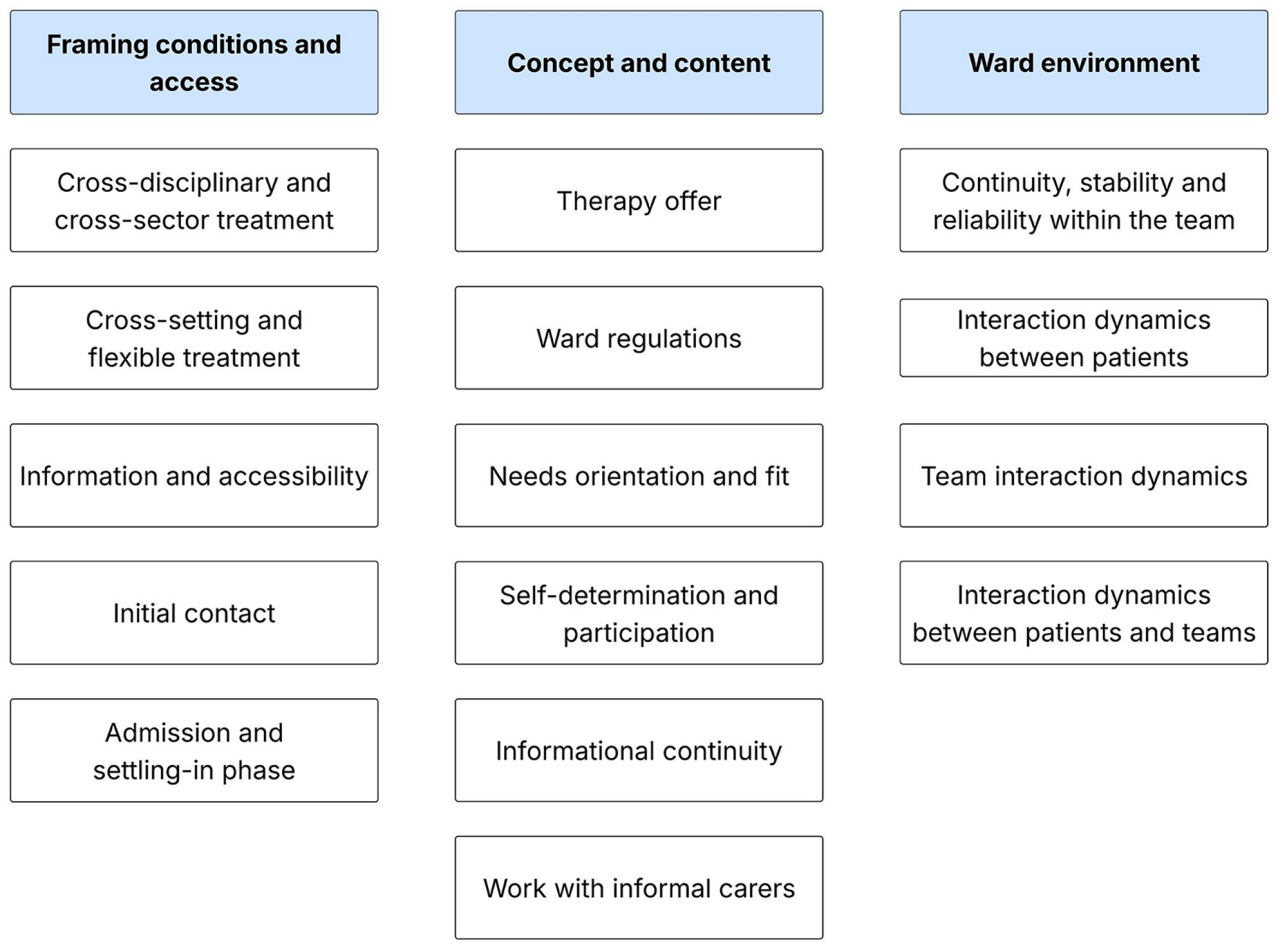
Qualitative findings: themes and subthemes

The qualitative results are presented thematically along themes and subthemes. Figure 2 provides an overview. The anchor quotes provide insight into the data collected. The abbreviations used refer to the socio-demographic characteristics of the respondents (App. Tab. 1).

### Framing conditions and access

The main category Framework conditions and access describes structural and procedural aspects that facilitated and secured access to the ADO as well as entry into treatment. This category includes Cross-disciplinary and cross-sector treatment, Cross-setting and flexible treatment, Information and accessibility, Initial contact and Admission and settling-in phase.

#### Cross-disciplinary and cross-sector treatment

Patients experienced individualized treatment adapted to their developmental stage only through the joint involvement of staff from CAP and AP:

*“I think they are trained for that. Some of them are from CAP and others are from AP. I think they do it very well. They treat us in an age-appropriate way*,* and it feels good the way it is.” (Patient*,* 21*,* f)*.

#### Cross-setting and flexible treatment

Patients perceived the possibility of switching quickly and without bureaucracy between inpatient, day hospital, and AOC settings as supportive of their recovery. This flexibility was especially valued in the context of stress testing, where changes in setting could also be reversed at short notice:

*“Without the AOC I would have had to discontinue treatment. This way I received the support I needed from the therapists I already knew*,* now in the outpatient instead of the inpatient setting.” (Patient*,* 20*,* f)*.

The AOC was also experienced as a suitable entry point into inpatient treatment. The flexible start on selected days facilitated contact with the ward and strengthened trust in the treatment concept:

*“I thought it was great that the therapists immediately noticed that day hospital treatment was not sufficient for my daughter and then admitted her as an inpatient right away. That way she got the help she needed.” (Mother*,* 43)*.

The option to return to the ADO in case of crisis after discharge was described as relieving:

*“The [therapists] always offered my daughter the option to turn to the ADO again whenever necessary. In acute situations*,* it was even possible to have a phone call with a therapist. I thought it was great that the children were not left alone after discharge.” (Mother*,* 42)*.

The coexistence of patients in different treatment settings on the ward was described as enriching:

*“I thought it was great that patients with AOC status were also present on the ward. That way*,* good conversations could take place*,* which improved the atmosphere on the ward.” (Patient*,* 18*,* m)*.

#### Initial information

Most patients did not learn about the ADO through their own prior experience in the health care system but were informed about it by therapists, parents, or peers:

*“My outpatient psychotherapist recommended the ADO to me. She told me that other patients of hers had had good experiences with treatment there and encouraged me to apply for admission.” (Patient*,* 21*,* f)*.

#### First contact

An initial appointment at the ward facilitated entry into treatment. Patients were able to get to know the structures and staff in advance, which reduced barriers:

*“If this first information meeting at the ADO had not taken place*,* I probably would not have taken the spot there.” (Patient*,* 21*,* f)*.

#### Admission and acclimatization

The sponsorship and primary nursing systems facilitated orientation and arrival. The proactive guidance was experienced as supportive:

*“After the admission interview I was shown my room. My sponsor showed me around the entire ward. Afterwards my primary nurse introduced himself and explained how everything works here.” (Patient*,* 19*,* m)*.

### Concept and content

The main category Concept and content describes central therapeutic and conceptual features of care at the ADO as experienced by patients and parents. It includes the subthemes Therapy offer, Ward regulations, Needs orientation and fit, Self-determination and participation, Informational continuity and Work with informal carers.

#### Therapy offer

Patients described the therapeutic program as diverse and supportive of recovery. The individualized therapy plan provided structural orientation but required patients to organize their therapeutic sessions themselves. This demand for initiative was evaluated differently:

*“For your weekly treatment plan you have to show a lot of initiative. That is fine*,* but I would prefer to have a pre-prepared plan and only make small changes if something does not fit.” (Patient*,* 21*,* f)*.

Staff absences due to sick leave or vacation sometimes disrupted scheduled therapy, which was described as a hindrance to progress:

*“The only thing that bothered me a little was that sometimes there was not enough staff. That is a bit annoying when you actually want to make progress here*,* but sessions are canceled.” (Patient*,* 19*,* f)*.

Continuity and intensity of therapy were experienced differently. While some patients felt overwhelmed, others described a balanced treatment density. Overall, patients agreed that the ADO’s individualized therapy program allowed sufficient free time to process what was learned in therapy:

*“I think the number of therapy sessions was exactly right. You could always participate in something but still had time for yourself.” (Father*,* 47)*.

#### Ward regulations

Compared to CAP, patients described the care concept at the ADO as more open and autonomy-promoting. The less rigid implicit and explicit behavioral rules were perceived as guidelines rather than restrictions, with freedoms such as carrying smartphones and leaving the premises independently. While some patients found the rules appropriate, others wished for even fewer restrictions:

*“In CAP I was treated like a child. There were strict rules*,* and you could not take responsibility for yourself. And in my opinion*,* that is exactly what is very important for adolescents between 16 and 18. That is when you learn to become an adult. I never learned that*,* so now I am doing it here with the support of the ADO.” (Patient*,* 19*,* f)*.

#### Needs orientation and fit

The tailoring of programs to age- and development-related needs was perceived as appropriate. Differences in maturity within the group could be addressed through individualized offers:

*“Here at the ADO*,* much more attention is paid than in CAP or AP to ensuring that age-appropriate programs are available. And I think that actually works pretty well.” (Patient*,* 21*,* f)*.

The school program and the ADO class (Career guidance for patients who have successfully completed their schooling) were considered particularly helpful. These offers were perceived as valuable for life planning:

*“Above all*,* I thought the school was great. It is wonderful that they help the children here look for an apprenticeship or write applications. That is exactly the difference from adult care. I think that is really excellent.” (Mother*,* 42)*.

#### Self-determination and participation

Patients experienced opportunities to actively shape their treatment. Being included in decision-making processes provided a sense of security and strengthened self-efficacy:

*“I know that I can help shape my treatment plan here and that decisions are not made over my head like in CAP. That gives me security.” (Patient*,* 20*,* f)*.

#### Informational continuity

Transparent communication between staff and patients helped avoid misunderstandings. Continuous information-sharing within the team was perceived as professional and created a sense of safety:

*“The team communicated everything clearly and was transparent.” (Patient*,* 20*,* m)*.

From the respondents’ point of view, patient-related (information) exchange within the ADO team is implemented in such a way that informational continuity between employees is achieved. Patients appreciate that employees were informed about the issues they were treating and actively asked questions. This gave patients a feeling of security:

*“The therapists passed on important information well during handovers. Especially when one of the primary therapists was present in the morning shift*,* they would come up to you and ask directly. For example: ‘Hey*,* do you want to talk about it again? What happened yesterday?’ I thought that was good.” (Patient*,* 18*,* f)*.

#### Work with informal carers

Work with informal carers was offered by the team but was evaluated inconsistently. Parents wished for stronger involvement and more information to better support their children after discharge:

*“I would have liked to be invited to a meeting so that we would have known what to expect after discharge. We did not know how to deal with our child once they were back home.” (Mother*,* 42)*.

Patients, however, often rejected family meetings, as they perceived them as undermining autonomy:

*“If my therapist had said*,* ‘there are meetings with parents now*,*’ I would not have understood that. Because then I would feel like a little child again.” (Patient*,* 23*,* m)*.

### Ward environment

The main category Ward Environment describes the social and emotional climate at the ADO and its contribution to patients’ sense of safety, stability, and recovery. It includes continuity, stability, and reliability within the team, interaction dynamics between patients, Team interaction dynamics and Interaction dynamics between patients and team.

#### Continuity, stability, and reliability within the team

Patients described the staff at the ADO as reliable, open, and approachable at all times. This presence was experienced as stabilizing:

*“Last Friday evening I had an emotional breakdown*,* and then I went to one of my primary nurses. She talked with me about it and advised me what I could do. So*,* it is very helpful that there is always someone you can talk to about how you are feeling and what you need.” (Patient*,* 18*,* f)*.

The primary nursing system with fixed contact persons was experienced as structuring and provided emotional security:

*“I think it definitely would have been more chaotic if there had not been the primary nursing system. Having fixed contact persons brings a certain stability to the daily routine.” (Patient*,* 19*,* f)*.

#### Interaction dynamics between patients

Patients experienced the community on the ADO as supportive. Similar age and comparable life situations enabled mutual identification:

*“I think the environment here is just right. You have people your age with similar interests with whom you can have good conversations. Somehow the balance is right. I think it fits very well. You can support each other much better*,* the atmosphere is very pleasant*,* and that also helps me a lot.” (Patient*,* 18*,* m)*.

Parents also reported positive effects of patient exchange:

*“At first*,* I thought maybe it is not always good to see other people’s problems or to compare yourself. But in the end*,* it is actually quite good to have this exchange of experiences with others.” (Mother*,* 53)*.

#### Team interaction dynamics

According to patients, the way team members interacted with each other had a positive effect on the ward atmosphere. Staff were perceived as role models:

*“I think the behavior of the therapists on the ward has an extreme effect on us. They create a positive environment by getting along well with each other*,* being active*,* and modeling that. You can orient yourself to that.” (Patient*,* 21*,* f)*.

#### Interaction dynamics between patients and team

Patients described their relationship with the team as trusting. Feeling accepted and understood encouraged emotional openness:

*“For about three or four weeks now*,* I have actually been able to let my feelings run completely free. The trust between the therapists*,* the primary nurses*,* and me is definitely there. That is because you can feel that there are people here who really want to help you.” (Patient*,* 19*,* f)*.

## Discussion

A particularly positive aspect highlighted was the multiprofessional composition of the team from CAP and AP, which enabled developmentally appropriate and individually tailored treatment. The flexible treatment concept, with fluid transitions between inpatient, day hospital, and AOC, was experienced as helpful for adapting the course of treatment to patients’ recovery progress. The possibility of switching between settings at short notice or receiving renewed support in acute situations after discharge was perceived as an important resource. Access to the ADO was facilitated by low-threshold information and contact opportunities. Initial personal meetings and the chance to get to know the ward and team beforehand lowered barriers to treatment entry. Admission and acclimatization were supported by established structures such as a sponsorship system and fixed contact persons, which provided orientation, safety, and emotional relief, especially during the initial phase.

Central to the experiences were perceptions of the scope, accessibility, and fit of therapeutic offerings, the handling of rules and autonomy, the orientation toward developmental needs, and the experience of participation and transparent communication. Family involvement and the importance of individualized treatment structures were also addressed. Taken together, these aspects portray the ADO as a care model focused on self-responsibility, developmental support, and therapeutic fit for adolescents.

The reliability and availability of the multiprofessional team, as well as the continuity provided by fixed contact persons, were described as particularly beneficial for creating a healing atmosphere. Interactions among patients were characterized by strong mutual support and a sense of connectedness, fostered by the age-homogeneous composition of the group. The positive team culture among staff was also perceived as a model and contributed to the quality of the social climate. Overall, interactions between patients and staff were marked by trust, openness, and mutual respect, which promoted emotional relief and therapeutic effectiveness. In this case, the effects that have been demonstrated in other studies become evident [46, 47].

The specialized treatment program for adolescents at the Vitos Klinik Südhessen was consistently perceived as a particularly suitable form of care. All patients stated that they would recommend the ADO, or that they had been referred there based on recommendations from external providers or former patients. Such word-of-mouth recommendations point to the high quality of treatment at the ADO. Patients reported feeling well cared for and noted improvements in their symptoms.

The specific focus on adolescents aged 16 to 25 enabled strong mutual identification and a supportive group dynamic. These findings reflect central developmental psychology insights, which emphasize the role of peers during late adolescence for emotional security and social development. Likewise, studies show that age-homogeneous settings facilitate psychosocial maturation and strengthen the sense of belonging [48, 49]. When minors and adults are accommodated and treated together, the protection of children and young people also plays a central role. Issues such as closeness and distance are therefore communicated openly as part of the treatment. Physical contact is prohibited. In order to create a low threshold, an anonymous reporting office is provided, but this is rarely used due to the positive relationships between practitioners and patients. The treatment team focuses on transparency and communication, also in line with Safewards. In addition, ADO patients are defined as an age group. Ward allocation is therefore not based solely on age, but on the patients’ level of development, which makes special protection obsolete.

The positive evaluation of cross-setting and modular care, including acute outpatient, day hospital, and inpatient components, underlines the relevance of individualized treatment options. This flexibility allowed therapeutic interventions to be adapted to individual maturity and stress levels, which represents a key success factor often missing in conventional transition models. Particularly during transitional phases, such staged and reversible treatment appears essential for preventing chronicity and service discontinuities. Therapeutic relationship building at the ADO was further strengthened by Safewards interventions and a structured primary nursing system [42]. The consistently participatory and respectful attitude of staff, combined with familiarity and trust within the ward, fostered the development of therapeutic relationships that provided orientation, enhanced coping ability, improved difficult behaviors, and contributed to patients feeling safer and more comfortable during their stay. These results confirm the importance of relationship-oriented milieu concepts in ADO.

Taken together, these structural characteristics position the ADO - depending on the local healthcare context - closer to a psychotherapeutically oriented unit within the acute care spectrum rather than to a high-acuity ward. Patients who pose an acute risk to themselves or others are temporarily transferred to specialized acute settings and return to the ADO after stabilization. Consequently, the direct transferability of the presented model to wards with high rates of acute crises and compulsory treatment may be limited. At the same time, the low-threshold and flexible services provided by the ADO may help prevent compulsory treatment in some cases. Crucially, the global treatment budget implemented in the Riedstadt model provided the financial flexibility required to initiate and sustain the structural and procedural transformations essential for the ADO, enabling cross-setting care, flexible resource allocation, and new care pathways beyond traditional inpatient logic. While the broader transformative effects of global treatment budgets on psychiatric care have been extensively examined elsewhere [36, 37, 50], this framework appears to be a key enabling condition for the development and scalability of adolescent-specific care models such as the ADO.

A clear imbalance emerged between the perspectives of patients and parents. This highlights the challenge of balancing adolescents’ autonomy with parental involvement in treatment. While parents often wished for more active involvement, many patients emphasized autonomy and self-determination, particularly in the context of detachment from parents. Parents’ limited participation in treatment was often due to patients’ explicit wishes, which also influenced their responses in interviews. This ambivalent dynamic reflects typical processes of adolescence and underlines the need for differentiated, voluntary forms of family-oriented work. The data also indicated the importance of peer environments as relevant support systems.

### Implications for research

Future studies should more closely examine the long-term benefits of the investigated model, including relapse rates, educational and occupational trajectories, and subjective quality of life after treatment. In addition, in-depth analyses of the model’s sensitivity to diversity are needed to evaluate its transferability to different target groups, such as queer youth, individuals with migration backgrounds, or adolescents in precarious life situations.

A key research desideratum is the development of a systematic review and a national care atlas for adolescent-specific psychiatric services. At present, no nationwide overview exists of existing structures, model projects, or specializations in the transition area between CAP and AP. The lack of transparent information hampers scientifically informed service planning. Mapping existing initiatives could help identify structural gaps, make good-practice models comparable, and address regional differences with an evidence base [21].

To standardize and further develop existing models, a catalog of criteria for adolescent-specific psychiatric care must be conceptually developed. This should be evidence-based and define the structural, personnel, and therapeutic elements necessary to meet the complex needs of individuals aged 16–25. Initial criteria can be derived from the EvAdoR study as well as international programs such as “Headspace” and “Foundry,” but these must be systematized through a consensus process [26, 27].

Building on such a catalog, a nationally adaptable implementation guide is required to support structured replication of successful models—such as the ADO Riedstadt—in other regions. Realistic evaluation designs and implementation studies can identify the conditions needed for sustainable implementation (e.g., legal frameworks, staffing structures, cooperation models) [51, 52].

To empirically test the actual effectiveness of adolescent-specific models compared with conventional transition programs, comparative studies are necessary. Current literature repeatedly describes the problems of abrupt transitions from CAP to AP, including relationship disruptions, interruptions in care, and rehospitalizations [20, 25]. Comparative study designs could assess the extent to which innovative models reduce these risks.

Furthermore, the mechanisms of specific components within the ADO—such as DBT-A, Safewards, the primary nursing system, and peer elements—should be investigated in more detail. The question of the optimal combination of inpatient, day hospital, and outpatient modules within a modular and flexible treatment pathway remains unanswered. Outcome-oriented mixed-methods studies are needed to develop evidence-based guidelines for the integration of these elements [45, 53].

International models demonstrate that digital interfaces in youth psychiatry can play an important role in destigmatization, aftercare, and early intervention [26]. Integrating digital tools into German models—for example, digital consultations, therapy apps, or platforms for coordinating multiprofessional providers—represents another central line of research.

### Implications for clinical practice

The positive perception of the familiarization phase and initial contacts underscores the importance of low-threshold services. These could be integrated more closely into existing care systems in order to facilitate access to adolescent-specific services. Schools, as central living spaces for young people, could be more closely involved as partners in prevention and intervention. School psychologists and teachers should be trained to recognize mental health issues at an early stage and initiate targeted support.

The combination of inpatient, day care, and outpatient services has proven to be an effective strategy. This model should be further developed and better networked regionally to enable individualized treatment courses. In rural or underserved regions in particular, digital solutions such as telemedicine could help to increase the reach and flexibility of services.

During the data evaluation period, the Vitos Clinic in Riedstadt implemented an outpatient clinic for adolescents. In addition to inpatient care, this enables individualized, disorder-specific treatment that takes adolescent-specific issues into account, even in the outpatient sector, and closes the gap in outpatient care.

### Strengths and limitations

This study represents the first comprehensive qualitative investigation of experiences with adolescent-specific psychiatric care from the perspectives of both patients and parents. A potential limitation is that the EvAdoR study was conducted at a single site and therefore explored only one concrete implementation of an ADO, which may restrict the generalizability of the findings to other regions. However, the data analysis was structured to identify generalizable aspects of ADOs that could be applied to other adolescent psychiatric care models. Participants were selected based on patient interest. It is possible that patients who had positive experiences with ADO were particularly likely to register. Self-selection bias or response bias cannot therefore be ruled out. Since these were semi-structured interviews and there was no standardized response logic, we do not report quantitative frequencies of individual responses or counts of positive or negative statements; the focus of the analysis is on patterns of meaning and themes, not on quantitative weighting.

Recruiting parents for qualitative interviews proved challenging, as the research team only had access to them through the patients. In many cases, patients refused to share their parents’ contact information. Nevertheless, satisfactory data saturation was achieved after interviewing seven parents. Patients’ reluctance to involve their parents was also reflected in the interviews, indicating that they were in a detachment process at the time of treatment and generally did not want parental involvement.

## Conclusions

The findings of this study highlight the importance of specialized, age-specific psychiatric care for adolescents. High patient satisfaction, evidenced by positive feedback and recommendations, speaks to the effectiveness of the ADO at Vitos Klinikum Riedstadt. A key success factor is age homogeneity, which fosters strong mutual identification and intensive exchange on developmentally relevant topics. This environment allows typical challenges such as insecurity, fear of embarrassment, and conformity pressure to be addressed within a protected setting.

The targeted consideration of specific needs—particularly regarding identity formation, autonomy development, and social competence—supports individualized therapy. The structured acclimatization phase and the implementation of Safewards interventions foster a safe treatment environment and the development of stable therapeutic relationships. Compared to traditional care models, the ADO offers a balanced relationship between predefined structures and individual freedom.

Despite challenges in involving parents, the findings suggest that flexible, family-oriented approaches could provide additional support. External factors such as the COVID-19 pandemic and the increasing influence of digital media highlight the urgent need for adolescent-specific interventions, including cooperation with schools to identify mental health problems at an early stage.

These results provide important starting points for future research and clinical practice projects aimed at ensuring the sustainability of treatment outcomes and continuously improving the quality of care.

## Data Availability

The qualitative data collected within the current study are not publicly available due to privacy restrictions but are available from the corresponding author upon reasonable request.

## Abbreviations

AOC: Acute outpatient care
ADO: Adolescent ward
DBT-A: Dialectical behavioral therapy for adolescents
AP: Adult psychiatry
CAP: Child and adolescent psychiatry
